# Mammary tumour development is dose-dependently inhibited by n-3 polyunsaturated fatty acids in the MMTV-neu(ndl)-YD5 transgenic mouse model

**DOI:** 10.1186/1476-511X-13-96

**Published:** 2014-06-11

**Authors:** Michael A Leslie, Salma A Abdelmagid, Kate Perez, William J Muller, David WL Ma

**Affiliations:** 1 Department of Human Health and Nutritional Sciences, University of Guelph, Animal Science/Nutrition Building, Room 342, 491 Gordon Street, N1G 2W1 Guelph, ON, Canada; 2 Molecular Oncology Labs, McGill University, Royal Victoria Hospital, Montreal, QC, Canada

**Keywords:** n-3 PUFA, Eicosapentaenoic acid, Docosahexaenoic acid, Mammary gland, MMTV-neu mice, Breast cancer, Tumour, Tumour multiplicity, Tumour volume, Phospholipids

## Abstract

**Background:**

Breast cancer is attributable to modifiable risk factors including the intake of dietary n-3 polyunsaturated fatty acids (PUFA). A key piece of evidence, yet to be addressed, that would demonstrate a causal relationship between n-3 PUFA and breast cancer, is a dose-dependent effect of n-3 PUFA on tumour outcomes. Thus, the objective of the present study was to determine whether n-3 PUFA reduces mammary gland tumor outcomes in a dose-dependent manner in female MMTV-neu(ndl)-YD5 transgenic mice, an aggressive model of human breast cancer.

**Methods:**

Harems were provided one of three experimental diets comprised of 0, 3 or 9% (w/w) menhaden fish oil containing n-3 PUFA. Female offspring were weaned onto the same parental diet and maintained on their respective diet for 20 weeks. Tumour onset, size and multiplicity were measured throughout the study. Fatty acid composition of mammary gland and tumours were determined by gas–liquid chromatography.

**Results:**

Tumour size was significantly (p < 0.05) reduced in a dose-dependent manner. n-3 PUFA were also incorporated in a dose-dependent manner; differential incorporation was observed for eicosapentaenoic and docosapentaenoic acids into mammary gland tissue, while docosahexaenoic acid was preferentially incorporated into tumours.

**Conclusion:**

Overall, the present study provides fundamental knowledge about the dose-dependent effect of n-3 PUFA on tumour outcomes in a pre-clinical model and also sheds light on the differential role of individual n-3 PUFA on tumour outcomes.

## Background

Breast cancer is more prevalent in Western countries, including Canada (2012 incidence rate was 99 in 100, 000 people) [1], than in Asian countries, such as Japan (2012 incidence rate was 44 in 100, 000 people) [2]. However, the difference in incidence rates between Western and Asian societies is diminished when Asian populations migrate to Western countries [3,4]. This suggests that environmental factors, including diet, may play a role in breast cancer development. The intake of n-3 polyunsaturated fatty acids (PUFA), which is high in Asian diets and low in Western diets, has been shown to be inversely correlated with breast cancer incidence [5-8], while n-6 PUFA consumption, high in Western diets, is associated with breast cancer incidence [4]. Recent reviews and meta-analyses support a role for n-3 PUFA in breast cancer prevention [9,10].

In rodent and in vitro models, n-3 PUFA have been shown to reduce neoplastic growths [11-15]. Previously, we have demonstrated the anti-tumourigenic effect of n-3 PUFA using the MMTV-neu(ndl)-YD5 mouse model. Intake of a 3% weight/weight (w/w) menhaden fish oil based diet reduced tumour volume and burden by ~30% in comparison to mice receiving a 10% (w/w) n-6 PUFA diet [14]. The MMTV-neu(ndl)-YD5 mouse model over-expresses the neu oncogene which is homologous with the human epidermal growth factor receptor 2 (HER2) gene found in humans. The over-expression of HER2 is estimated to be responsible for 20-25% of human breast cancer cases [15-17]. The incorporation of n-3 PUFA into cellular and tumour lipids is hypothesized to be an important mechanism by which n-3 PUFA elicit their anti-tumourigenic effects [13,18-20]. Other mechanisms include the competitive inhibition of arachidonic acid derived eicosanoids and the alteration of lipid rafts [21].

While previous studies have shown that consuming n-3 PUFA at a single dose has the ability to reduce tumour growth [14,18], to the best of our knowledge no one has investigated the dose-dependent effect of n-3 PUFA in experimental rodent models. This fundamental knowledge is lacking and highly important for substantiating the cause-effect relationship between n-3 PUFA exposure and breast cancer outcomes. Thus, the present study examined the effect of increasing levels of n-3 PUFA, 0%, 3% and 9% (w/w), on tumour onset, size, multiplicity and the incorporation of n-3 PUFA into phospholipids of tumours and their adjacent mammary glands using MMTV-neu(ndl)-YD5 mice.

## Results

### Body growth and puberty onset

Final body weights of mice on the 0% n-3 PUFA diet (27.6 ± 0.6 g) were not significantly different than those of mice fed a 9% n-3 PUFA diet (28.7 ± 0.7 g). However, mice fed either of the aforementioned diets had a significantly smaller final body weight than mice fed the 3% n-3 PUFA diet (31.8 ± 0.8 g, p < 0.05). Puberty onset was significantly (p < 0.05) delayed in mice fed either the 3% (30.6 ± 1.7 days) or 9% (30.1 ± 0.4) n-3 PUFA diets compared to mice fed the 0% n-3 PUFA diet (27.6 ± 0.5).

### Tumour latency and tumour free status

**Figure 1:**
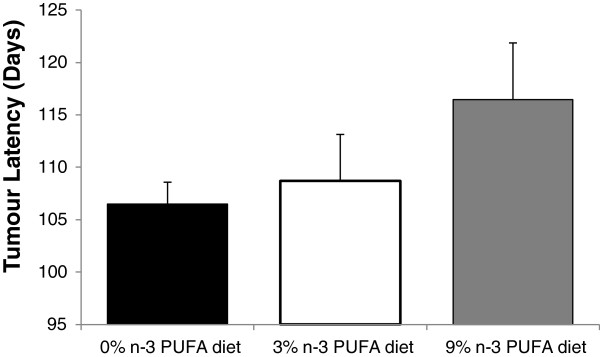
**The average tumour latency of mice fed a 0% (n = 16), 3% (n = 10) and 9% (n = 11) n-3 PUFA diet.** No significant difference existed between groups as determined by a one-way ANOVA.

Tumour latency was not significantly different between the 3 groups, but average tumour latency, defined as the average age at which the first tumour was identified, was delayed in mice fed both 3% n-3 PUFA (108.7 ± 4.1 days) and 9% n-3 PUFA (115.6 ± 3.9 days) relative to 0% n-3 PUFA (106.5 ± 3.2 days) fed mice (p = 0.21) (Figure 1).

**Figure 2:**
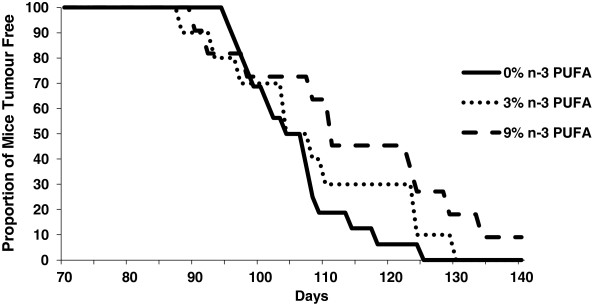
**The proportion of mice tumour free throughout the duration of the study fed a 0% n-3 PUFA diet (n = 16), 3% n-3 PUFA diet (n = 10), and 9% n-3 PUFA diet (n = 11).** More mice remained tumour free longer in 3% and 9% n-3 PUFA fed mice.

The median age at which tumours were palpable (T_50_) for the groups fed either the 0% or the 3% n-3 PUFA diets was 104 days, while the T_50_ for the group fed a 9% n-3 PUFA diet was 110.5 days (Figure 2).

### Tumour multiplicity

**Figure 3:**
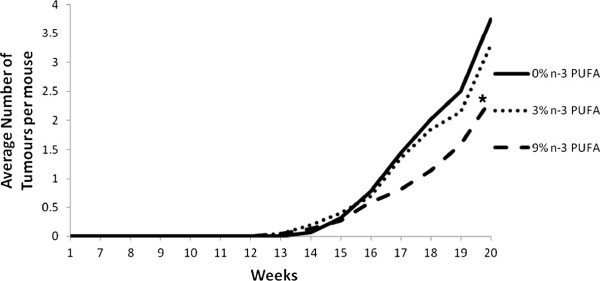
**The average number of tumours palpitated per dietary intervention throughout the course of the study in mice fed a 0% n-3 PUFA diet (n = 16), 3% n-3 PUFA diet (n = 10), and 9% n-3 PUFA diet (n = 11).** The asterisk indicates that mice fed a 9% n-3 PUFA diet had a significant reduction in tumour multiplicity in comparison to mice fed either of the other diets as determined by a repeated measures analysis (p < 0.05).

Repeated measures analysis demonstrated that mice fed the 9% n-3 PUFA diet had significantly reduced tumour multiplicity in comparison to mice fed either the 0% or 3% n-3 PUFA diet, beginning on days 115 and 122, respectively (p < 0.05) (Figure 3). This difference was maintained until the end of the study at 140 days. Final tumour multiplicity was reduced by 12% and 40% in the 3% and 9% n-3 PUFA fed mice, respectively, relative to the 0% n-3 PUFA fed controls.

### Tumour volume

**Figure 4:**
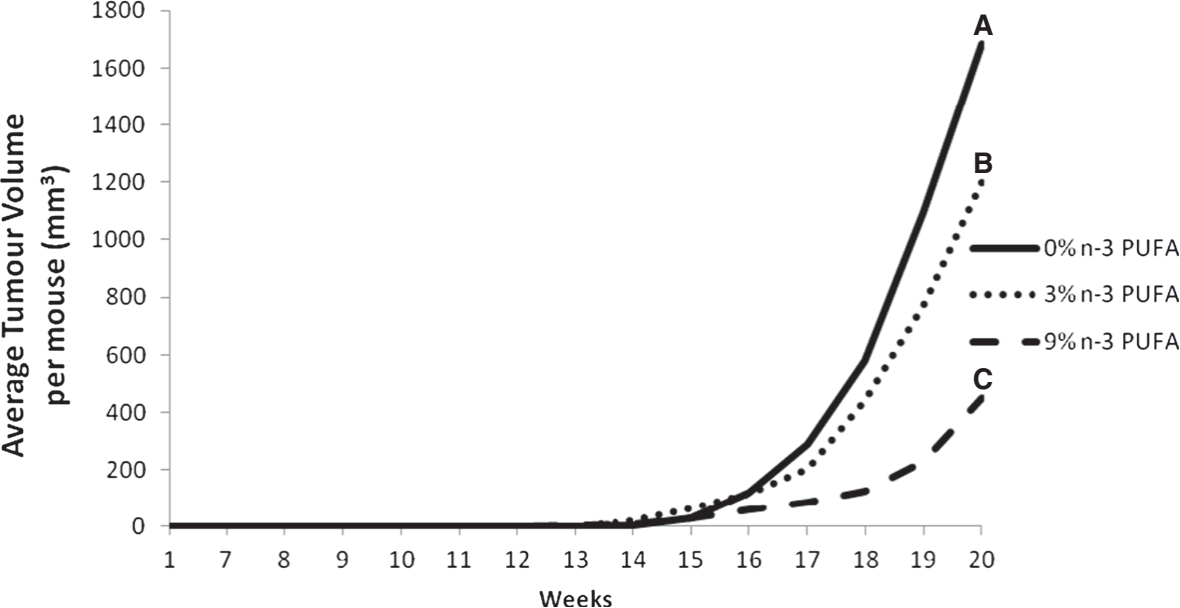
**The average tumour volume per dietary intervention over the duration of the study in mice fed a 0% n-3 PUFA diet (n = 16), 3% n-3 PUFA diet (n = 10), and 9% n-3 PUFA diet (n = 11).** Groups with a different letter indicate significantly different tumour volumes as determined by a repeated measures analysis (p < 0.05).

Repeated measures analysis demonstrated that average tumour volume began to diverge significantly at 117 and 120 days of age for mice fed a 9% n-3 PUFA diet in comparison to mice fed a 0% n-3 PUFA diet as well as a 3% n-3 PUFA diet, respectively (p < 0.05). While mice fed a 3% n-3 PUFA diet had significantly reduced average tumour volume by day 133 in contrast to mice fed the 0% n-3 PUFA diet (p < 0.05) (Figure 4). Final tumour volume was ~35% and 70% lower in the 9% n-3 PUFA fed mice relative to the 3% and 0% n-3 PUFA fed mice, respectively.

### Mammary gland phospholipid composition

The incorporation of n-3 PUFA into PC within the mammary gland was assessed to determine if the observed effects were due to altered fatty acid composition of the plasma membrane (Table 1). Compared to mice fed the 0% n-3 PUFA diet (n = 5), there was preferential incorporation of eicosapentaenoic acid (EPA, 20:5 n-3) and docosapentaenoic acid (DPA, 22:5 n-3) in mice fed a 3% n-3 PUFA diet and further elevated in mice fed a 9% n-3 PUFA diet (p < 0.05). Correspondingly, there was a significant (p < 0.05) dose-dependent decrease in arachidonic acid (AA, 20:4 n-6). The n-6:n-3 PUFA ratio also significantly decreased (p < 0.05) from 23.8 ± 8.5 in mice fed 3% n-3 PUFA (n = 4) to 3.4 ± 1.0 in mice fed the 9% n-3 PUFA diet (n = 5). Also, the ratio of PUFA: saturated fatty acids (SFA) within PC was significantly reduced (p < 0.05) in mice fed either a 3% or 9% n-3 PUFA diet (respectively, 0.6 ± 0.04 and 0.5 ± 0.05) in comparison to mice fed the 0% n-3 PUFA diet (1.0 ± 01). Similar effects were observed in the PE fraction (data not shown).

**Table 1:** Fatty acid composition of the phosphatidylcholine fraction within mammary glands adjacent to tumours

**Mammary gland PC fatty acids**	**Diet**		
	**0%**	**3%**	**9%**
LA (18:2n-6)	30.8 ± 2.1^a^	20.9 ± 2.3^b^	11.9 ± 1.6^c^
AA (20:4n-6)	8.3 ± 0.5^a^	3.8 ± 0.2^b^	7.9 ± 2.1^ab^
DPA (22:5n-6)	0.8 ± 0.1^a^	trace^b^	trace^b^
ALA (18:3n-3)	trace^a^	trace^a^	trace^a^
EPA (20:5n-3)	trace^a^	0.7 ± 0.1^b^	4.0 ± 0.8^c^
DPA (22:5n-3)	trace^a^	0.3 ± 0.4^ab^	1.9 ± 0.9^b^
DHA (22:6n-3)	trace^a^	trace^a^	trace^a^
Total n-6	41.4 ± 1.7^a^	25.2 ± 2.1^b^	20.3 ± 1.2^c^
Total n-3	0.03 ± 0.03^a^	1.1 ± 0.5^b^	6.0 ± 1.5^c^
n-6/n-3 ratio	1292.3^a^	23.8 ± 8.5^b^	3.4 ± 1.0^c^
Total SFA	43.6 ± 1.1^a^	45.9 ± 0.4^ab^	52.6 ± 0.9^b^
Total MUFA	14.1 ± 1.7^a^	23.4 ± 2.0^a^	21.0 ± 1.9^b^
Total PUFA	41.7 ± 1.3^a^	26.4 ± 2.6^b^	26.4 ± 1.4^c^
P/S Ratio	1.0 ± 0.1^a^	0.6 ± 0.04^b^	0.5 ± 0.05^c^

n = 4 for the 3% n-3 PUFA diet, n = 5 for both the 0% n-3 and 9% n-3 PUFA dietary interventions. Values in the same row not sharing the same superscript are significantly different from one another as determined by a one-way ANOVA (p < 0.05).

### Tumour phospholipid content

There were significant differences in the fatty acid composition of the tumor PC fraction between groups (n = 5 for mice fed either the 0% or the 3% n-3 PUFA diet, n = 4 for mice fed the 9% n-3 PUFA diet) (Table 2). A significant (p < 0.05) dose-dependent reduction was observed for linoleic acid (LA, 18:2 n-6) and AA in tumours of mice fed increasing levels of n-3 PUFA in comparison to mice fed 0% n-3 PUFA (p < 0.05). There was also a dose-dependent increase in EPA accumulation into tumours of mice fed increasing levels of n-3 PUFA in comparison to mice fed 0% n-3 PUFA (p < 0.05). DHA accumulation was only significantly greater in mice fed the 9% n-3 PUFA diet in comparison to mice fed 0% n-3 PUFA (p < 0.05). A significant dose-dependent reduction (p < 0.05) in tumour n-6:n-3 PUFA ratio was observed in both the 3% and 9% n-3 PUFA diets (9.5 ± 1.1 and 1.7 ± 0.1, respectively) in comparison to mice fed the 0% n-3 PUFA diet (24.6 ± 6.8). The ratio of PUFA: SFA was observed to be significantly reduced (p < 0.05) in mice fed a 9% n-3 PUFA diet (0.5 ± 0.03) compared to mice fed the 0% n-3 PUFA diet (0.87 ± 0.17). Similar effects were observed in the PE, PS and PI fractions (data not shown).

**Table 2:** Fatty acid composition of tumour phosphatidylcholine

**Tumour PC fatty acids**	**Diet**		
	**0%**	**3%**	**9%**
LA (18:2n-6)	10.1 ± 1.2^a^	13.9 ± 0.4^b^	5.1 ± 0.4^c^
AA (20:4n-6)	16.5 ± 1.3^a^	10.9 ± 0.6^b^	6.2 ± 0.3^c^
DPA (22:5n-6)	0.9 ± 0.2^a^	0.03 ± 0.02^b^	0.1 ± 0.1^b^
ALA (18:3n-3)	0.1 ± 0.1^a^	0^a^	0.01 ± 0.01^a^
EPA (20:5n-3)	0.1 ± 0.1^a^	0.8 ± 0.1^b^	3.1 ± 0.2^c^
DPA (22:5n-3)	0.2 ± 0.04^a^	0.8 ± 0.1^b^	1.3 ± 0.6^ab^
DHA (22:6n-3)	1.0 ± 0.2^a^	1.5 ± 0.3^a^	2.7 ± 0.2^b^
Total n-6	33.0 ± 2.3^a^	28.4 ± 0.6^a^	12.5 ± 0.9^b^
Total n-3	1.3 ± 0.3^a^	3.0 ± 0.3^b^	7.2 ± 0.4^c^
n-6/n-3 ratio	24.6 ± 6.8^a^	9.5 ± 1.1^b^	1.7 ± 0.1^c^
Total SFA	39.3 ± 1.3^a^	41.5 ± 0.4^a^	42.1 ± 0.9^a^
Total MUFA	26.4 ± 2.2^a^	26.2 ± 0.7^a^	38.1 ± 1.0^b^
Total PUFA	34.3 ± 3.3^a^	31.4 ± 0.7^a^	19.7 ± 0.6^b^
P/S Ratio	0.9 ± 0.2^a^	0.8 ± 0.03^a^	0.5 ± 0.03^b^

n = 4 for the 9% n-3 PUFA diet, n = 5 for both the 0% n-3 and 3% n-3 PUFA diets. Values in the same row not sharing the same superscript are significantly different from one another as determined by a one-way ANOVA (p < 0.05).

## Discussion

The present study addresses a fundamental question of causality by examining the dose response effect of n-3 PUFA on tumour outcomes in a pre-clinical model of breast cancer. Epidemiological studies to date only provide correlative evidence in support of a beneficial effect of n-3 PUFA in reducing the risk of developing breast cancer [10]. Thus, a major gap remaining is demonstrating a cause-effect relationship in human or preclinical models [22]. To the best of our knowledge a fundamental dose-dependent study of n-3 PUFA in breast cancer has not been previously reported. Thus, it is unknown whether effects at higher doses plateau or potentially cause harm. The results of this study provide evidence that the intake of n-3 PUFA, in a dose dependent manner results in a corresponding increase in n-3 PUFA in tumour phospholipids associated with a reduction in tumour outcomes.A key attribute of cause-effect relationships is demonstrating dose dependency. Multiple parameters in this study suggest a beneficial dose-dependent relationship across the 0%, 3% and 9% (w/w) levels of n-3 PUFA and tumour outcomes. On average, tumour latency was delayed by 2% and 9% in mice fed 3% and 9% n-3 PUFA diets, respectively (Figure 1). On average, the majority of mice were tumour free longer throughout the later time points in the 3% and 9% n-3 PUFA fed groups when compared to the 0% n-3 PUFA fed group (Figure 2). Tumour multiplicity was significantly (p < 0.05) decreased at the highest dose of 9% n-3 PUFA (Figure 3). Tumour volume was significantly (p < 0.05) and dose-dependently decreased at all levels of treatment (Figure 4).

The current study showed a greater reduction in final tumour volume in mice fed a 9% n-3 PUFA diet in comparison to findings of previous studies in MMTV-neu mice [14,15]. Yee et al. (2005) fed mice either an 11% (w/w) corn oil diet (enriched in n-6 PUFA) or a 10% (w/w) menhaden fish oil diet beginning at 7–8 weeks of age for the duration of a 61 week study [15]. The study utilized the MMTV-neu mouse model, a less aggressive model than the one utilized in the current study. These investigators showed that a diet supplying 22.5% of total energy (10% w/w) from menhaden oil resulted in a 30% reduction in tumour volume [15]. In contrast, a similar amount of energy provided by lifelong exposure to a 9% n-3 PUFA diet in the present study resulted in a 70% reduction in final tumour volume. These observations suggest a potential benefit of long term exposure to n-3 PUFA from conception through in utero development, lactation, weaning, puberty and adulthood confers added protection against mammary tumour development. This is supported by previous studies demonstrating that the timing of exposure may influence future cancer risk [11,23].

In regards to physiological relevance, the 9% n-3 PUFA diet provided mice with 22% of total calories from fat, but of greater relevance this diet provides 6.7% of the total daily energy requirements from EPA and DHA, or the equivalent of 11 g/day in humans (assuming the average human female consumes 2000 kcal/day). In humans the consumption of 267 g of fish per day will provide approximately 11 g of EPA and DHA, or alternatively the equivalent of 3 servings of an average 85 g salmon steak [24]. While the intake of 11 g of EPA and DHA per day may seem unrealistic, a study of 48 women demonstrated that intake of up to 9 capsules of marine based n-3 PUFA, providing up to 7.56 g of DHA + EPA a day, for 6 months was well tolerated by participants [25]. The prescribed dosages produced similar levels of compliance among participants and a dose response accumulation of EPA and DHA was observed in serum and breast adipose tissue [25]. In contrast, the equivalent quantity of EPA and DHA from the 3% n-3 PUFA diet is achievable in humans through the consumption of 3.4 g of EPA/DHA a day. Alternatively, the balance of n-6 to n-3 PUFA is another way to characterize the physiological relevance of the diets used in the present study. In Australia, the ratio of n-6 to n-3 PUFA in an individual’s diet ranges from 2 to 80, which is similar to the variance of this ratio used in the present study [26].

The anti-tumourigenic properties of n-3 PUFA are largely related to their ability to incorporate into the plasma membrane of target tissues and alter membrane-protein and subsequently protein-protein interactions [20]. The observed dose-dependent effects of a reduced n6:n-3 PUFA dietary intake were associated with a dose-dependent change in the fatty acid profile reflected in a decreased n-6:n-3 PUFA ratio, driven by increases in n-3 PUFA, mainly EPA, DPA and DHA, and a concomitant decrease in the n-6 PUFA, AA. These direct measurements importantly confirm that the bioactive components of the diet, n-3 and n-6 PUFA, are targeted to the mammary gland and tumour of interest, which coincided with reductions in tumour burden observed in the mice. Marine-based oils contain multiple n-3 PUFA, but the field has focused primarily on the study of EPA and DHA. However, the present study observed significant and appreciable incorporation of another n-3 PUFA, docosapentaenoic acid (DPA, 22:5 n-3) (Table 2). The biological role of DPA is not known, but one previous study noted a similar observation [27]. Thus, DPA deserves more attention in future studies regarding cancer. This is highly relevant given that DHA was only preferentially incorporated into tumour phospholipids but not into mammary gland phospholipids (Tables 1 and 2). While speculative, these observations suggest differential role of individual n-3 PUFA along the cancer trajectory. Further research is required to better define the interplay between individual and combinations of n-3 PUFA on tumour outcomes.

The present study did not examine mechanisms of action, but the change in membrane fatty acid composition is important as n-6 and n-3 PUFA compete for the same eicosanoid enzymes. The observed anti-tumourigenic effects of the diets utilized in this study may be due to a dose-dependent increase in n-3 PUFA consumption, a dose-dependent reduction in n-6 PUFA intake or a combination of the two with a dose-dependent reduction in the n-6:n-3 PUFA dietary ratio. Prostaglandins, leukotrienes and thromboxanes derived from AA promote inflammation and cellular proliferation; however, when they are derived from n-3 PUFA they promote apoptosis and inhibit cell proliferation [28]. Additionally, n-3 PUFA have been found to elevate pro-apoptotic factors including caspases 9 and 3, Bcl2, and reactive oxygen species, while also reducing DNA synthesis, cellular proliferation and the integration of growth factor receptors into the plasma membrane [18,19]. Changes in membrane composition were observed in both mammary and tumour tissue, but it is likely that the change in tumour composition is most relevant in explaining the anti-tumourigenic effects of n-3 PUFA. The control diet utilized in this study contained 10% (w/w) safflower oil which is abundant in LA and scarce in other n-6 PUFA. The n-6 and n-3 families of fatty acids refer to many structurally similar molecules which may have different biological effects. Thus, in this study, we can only conclude that in comparison to an LA rich diet, a fish oil diet rich in EPA and DHA is protective against tumour development.

Pubertal onset has been linked to breast cancer as earlier menarches place females at a greater risk for breast cancer [29]. In the present study, the onset of puberty in the mice was significantly (p < 0.05) delayed by ~3 days in mice fed either the 3% or 9% n-3 PUFA diets as compared to 0% n-3 PUFA fed mice. Mechanistically, n-3 PUFA may influence circulating estrogen levels, a key regulator of puberty [11]. This observation suggests further investigation on the role of n-3 PUFA in regulating hormonal influences on puberty and cancer. Furthermore, this observation supports growing evidence that critical periods of early development such as puberty have important influences on future cancer risk [23,30,31].

In conclusion, the present study contributes importantly to addressing an important gap in our fundamental understanding of the causal role for n-3 PUFA in breast cancer prevention.

## Methods

### Animals, diets and genotyping

Mice were housed in ventilated cages in a humidity and temperature controlled environment on a 12 hour light-12 hour dark cycle for the duration of the study. MMTV-neu(ndl)-YD5 mice on an FVB background were obtained from Dr. William Muller at the University of McGill. Harems consisted of one male heterozygous MMTV-neu(ndl)-YD5 mouse and three female FVB mice yielding progeny with a wildtype or a heterozygous genotype. Harems were fed a modified AIN93G diet (Research Diets Inc.) ad libitum containing 10% fat (w/w) providing 22% of the mouse’s total daily energy requirements. Mice received diets containing n-3 PUFA with different amounts (% w/w) of menhaden fish oil. Diets comprised either 0% n-3 PUFA (10% w/w safflower oil (rich in n-6 PUFA), n = 16); 3% n-3 PUFA (7% safflower oil and 3% menhaden oil, n = 10); or 9% n-3 PUFA (1% safflower oil and 9% menhaden oil, n = 11). The group receiving a diet containing 0% n-3 PUFA were used as a control as this is representative of a typical n-6 PUFA enriched, Western style diet. Offspring were weaned at 3 weeks of age and genotyped as described previously [14]. Female MMTV-neu(ndl)-YD5 transgenic offspring were maintained on their parental diet while males and wildtype females were euthanized post-weaning. Thus, female offspring received the same diet throughout life, from in utero until termination at 20 weeks of age. All experimental procedures were approved by the institutional animal care committee (University of Guelph).

### Phenotypic measurements

Post-weaning, female mice were checked daily for vaginal opening to determine the onset of puberty. The body weights of mice were measured weekly from 3 to 20 weeks of age on an electronic scale. Beginning at week 12, mice were palpated three times a week to detect the presence of new mammary gland tumour formations. When a tumour was detected, biweekly measurements were taken for the duration of the study. Tumours were measured along the sagittal (length) and transverse (width) planes with the use of electronic calipers and tumour volume was calculated using the formula [(length)•(width)^2^]/2.

### Euthanization and tissue collection

When mice reached 20 weeks (140 days) of age a vaginal smear was taken by flushing the vagina with 30 μL of a phosphate buffer saline solution. The solution was then placed on a glass slide and viewed under a Nikon Eclipse TS100 microscope for estrus cycle classification. Mice were identified as in proestrus, estrus, metaestrus or diestrus cycle by cell characteristics [14]. Mice in proestrus, estrus or metaestrus were euthanized by carbon dioxide asphyxiation. Euthanization of mice in diestrus was delayed in order to control for hormonal fluctuations which may impact cell proliferation profiles. For ethical reasons mice which lost more than 20% of their body weight or possessed tumours which exceeded either 17 mm in length/width or more than 5000 mm^3^ in volume were euthanized prior to the 20 week time point.

The mouse pelt, with mammary glands (MGs) and tumors attached, was removed to allow for final tumour volume measurements via electronic calipers, and to remove the 4^th^ and 5^th^ MGs as well as all tumours. The tumours and MGs were excised and snap-frozen in liquid nitrogen.

### Fatty acid analysis

Lipids were extracted from the 4^th^ and 5^th^ MGs (n = 5 for the 0% n-3 PUFA and the 9% n-3 PUFA diets, n = 4 for the 3% n-3 PUFA diet) and tumour tissues (n = 5 for the 0% n-3 PUFA and the 3% n-3 PUFA diets, n = 4 for the 9% n-3 PUFA diet) via the Folch Method [32] and separated into individual phospholipid classes by thin-layer chromatography (TLC). In brief, the entirety of a dissected MG adjacent to a tumour was homogenized on ice in 0.1 M KCl, added to 2:1 CHCl_3_: MeOH, vortexed and incubated overnight at 4°C to facilitate lipid extraction. The same technique was utilized for tumour tissue; however, only 0.1 g of the tissue was homogenized. The following day samples were centrifuged at 357 × g for 10 minutes, the chloroform layer was then collected and dried down under a gentle stream of nitrogen on a warm heating block. Dried down lipids from tumour tissues were reconstituted in chloroform to a concentration of 10 mg/ml. Samples from MGs were reconstituted in 200 μL of chloroform.

Lipids were spotted on H-plates (EMD Chemicals #5721-7) which were activated by incubating the plates in an oven for an hour at 100°C. Spotted H-plates were then placed in a thin layer chromatography tank containing 30 ml chloroform, 9 ml methanol, 25 ml 2-propanol, 6 ml 0.25 M KCl, and 18 ml triethylamine. The plate was then lightly sprayed with 0.1% (w/v) ANSA (Fluka #GA12046) before being visualized under UV light. Bands corresponding to phosphatidylcholine (PC), phosphatidylethanolamine (PE), phosphatidylinositol (PI) and phosphatidylserine (PS) were collected from tumour samples while only PC and PE bands were visible and collected from MG samples.

Methylation was performed by adding 2 ml of hexane and 2 ml of 14% BF_3_-MeOH (Sigma B1252) to the samples and incubating them at 100°C for 90 minutes. Following methylation, 2 ml of double distilled H_2_O was added to the samples and the solution was immediately vortexed for 30 seconds to halt methylation. Samples were centrifuged for 10 minutes at 357 × g, the hexane layer was collected and dried down under nitrogen before reconstitution in 2 ml of hexane. Fatty acid methyl esters were analyzed using a gas chromatography system (Agilent Technologies 7890A). Fatty acid composition was expressed as a percentage of total fatty acids.

### Statistics

SAS v9.1 was utilized for all statistical analyses. A one-way ANOVA was conducted to determine differences in final body weights, puberty onset, tumour latency, final tumour volume, final tumour multiplicity and fatty acid compositions of tumour and MG tissues. The Kruskal-Wallis test was performed for data that was not normally distributed. A repeated measures analysis was utilized to detect differences in tumour volume and multiplicity between diets over the duration of the 20 week study. A p < 0.05 was considered significant.

## Abbreviations

PUFA: Polyunsaturated fatty acids; MMTV: Mouse mammary tumour virus; HER2: Human epidermal growth factor receptor 2; w/w: Weight/weight; MG: Mammary gland; ALA: Alpha-linolenic acid; EPA: Eicosapentaenoic acid; DPA: Docosapentaenoic acid; DHA: Docosahexaenoic acid; LA: Linoleic acid; AA: Arachidonic acid; TLC: Thin layer chromatography; PC: Phosphatidylcholine.

## Competing interests

The authors declare that they have no competing interests.

## Authors’ contributions

ML, SA, KP and DM were responsible for the experimental design. ML, KP conducted the experiments, ML analyzed the data and drafted the manuscript. ML, SA, DM revised the manuscript. WM provided the strain of mice utilized in the experiment. All authors read and approved the final manuscript.
